# Reduction and Elimination of Humic Acid Fouling in Air Sparged Membrane Distillation Using Nanocarbon Immobilized Membrane

**DOI:** 10.3390/molecules27092896

**Published:** 2022-05-01

**Authors:** Mitun Chandra Bhoumick, Sagar Roy, Somenath Mitra

**Affiliations:** Department of Chemistry and Environmental Science, New Jersey Institute of Technology, Newark College of Engineering, University Heights, Newark, NJ 07102, USA; mb777@njit.edu (M.C.B.); sagar.roy@njit.edu (S.R.)

**Keywords:** air sparging, membrane fouling, carbon nanotubes, hydrophobic membrane and evaporation efficiency

## Abstract

In this paper, we present the treatment of humic acid solution via carbon nanotube immobilized membrane (CNIM) distillation assisted by air sparging (AS). Carbon nanotubes offer excellent hydrophobicity to the modified membrane surface and actively transport water vapor molecules through the membrane to generate higher vapor flux and better rejection of humic acid. The introduction of air sparging in the membrane distillation (MD) system has changed the humic substance fouling by changing the colloidal behavior of the deposits. This modified MD system can sustain a higher run time of separation and has enhanced the evaporation efficiency by 20% more than the regular membrane distillation. The air sparging has reduced the deposition by 30% in weight and offered lesser fouling of membrane surface even after a longer operating cycle. The water vapor flux increased with temperature and decreased as the volumetric concentrating factor (VCF) increased. The mass transfer coefficient was found to be the highest for the air sparged—carbon nanotube immobilized membrane (AS-CNIM) integrated membrane distillation. While the highest change in mass transfer coefficient (MTC) was found for polytetrafluoroethylene (PTFE) membrane with air sparging at 70 °C.

## 1. Introduction

NOM (natural organic matter) is mainly composed of humic substances and is known to contribute color to the water [1,2]. These substances are originated from the degradation of plants and micro-organisms either by chemical or biological pathway [3,4]. In terms of chemical property, humic substances are amphipathic and show both hydrophobic and hydrophilic nature [5,6,7]. As a result, NOMs removal is important to ensure water quality. To deal with this problem membrane-based filtration has been used to treat NOMs [2,8]. The filtration process is limited by the high fouling nature of humic substances in membrane surface and pores. Apart from filtration, high concentration of humic acid tends to foul pressure-driven reverse osmosis systems and also persists in the case of forward osmosis fouling; in fact, the humic acid fouling is prompted by reverse ionic draw solute flux.

Membrane distillation (MD) is a thermally driven membrane process that utilizes vapor pressure difference as the driving force across a hydrophobic membrane [9,10,11]. The hydrophobic character prevents wetting of the membrane surface and pore condensation during vapor permeation. Several types of membrane distillation configurations have been reported and these include direct contact membrane distillation, sweeping gas membrane distillation, air gap membrane distillation, and vacuum membrane distillation [12,13,14,15,16]. Recently we have reported the development of air sparged membrane distillation for the treatment of high fouling saline water [11]. Here, air sparging synergistically enhanced the permeate flux, mass transfer coefficients, and altered the colloidal behavior of deposits on the membrane surface. Another important development is nanocarbon immobilized membrane that uses carbon nanotube, graphene oxide, and reduced graphene oxide on the commercial membrane surface to enhance the hydrophobicity [9,11,17,18,19,20,21]. These nanocarbon materials, upon coating over the membrane surface, facilitated the diffusion of water vapor. The defects and functionalities on the membrane surface act as the active sites during permeation process [22,23,24].

Fouling is an important consideration in MD where adsorption in pores and plugging are the major fouling mechanisms [23,25,26,27,28]. The propagation of fouling leads to the lowering of hydrophobicity and wetting of the membrane that induces passing of the liquid through the membrane then the vapor molecules [3,29,30]. Fouling also leads to temperature and concentration polarization and decrease in flux, thus lowering the thermal efficiency of the MD process. Therefore, the mitigation in fouling in the presence of humic acid is an important consideration for MD. Fouling reduction in MD has been carried out by changing the flow regime across the membrane surface [31], use of antiscalants [20], and regular cleaning. We have also shown that immobilization of carbon nanotubes can reduce fouling in CaSO_4_ and CaCO_3_ contained water where the CNTs (carbon nanotubes) serve a screen that prevent the deposition of large particles [20,32,33].

In case of humic acid fouling, the macromolecules that clog the membrane pores eventually form a layer that lower water vapor mass transfer coefficient by layer resistance. The deposition of humic acid over the membrane surface could also be mediated by the presence of divalent ions. For example, calcium ions can form complex with NOM and precipitate over the membrane surface [34,35,36]. Several studies have been carried out to investigate humic acid fouling using hydrophobic membrane for different membrane configurations. Nanocarbons and metal organic frameworks have been used to modify the membrane surface [2,7,37]. Membrane and process modifications have been shown to effect both the flux and fouling [26]. Here, carbon nanotubes are used for membrane modification and sparging air for process modification. We anticipate that air sparging could potentially reduce the humic acid fouling during membrane distillation process.

Carbon nanotubes immobilized on the PTFE membrane surface change the dynamics by rendering active diffusion sites in the membrane [38,39,40,41]. The CNTs adsorbs the HA macromolecules, which upon the exposure of air sparging desorbs and cleans off. The surface modification of the membrane keeps the PTFE membrane unexposed to the HA molecules and acts as a screen. Once the layer is broken down, the HA layer can be cleaned by bubbles upon exerting shear stress.

The objective of this study is to investigate the reduction in fouling via AS-MD in presence of humic acids. Another objective is to study the use of carbon nanotube immobilized membrane in reducing humic acid fouling.

## 2. Materials and Methods

### 2.1. Chemicals

Humic acid sodium salt 45–70% was purchased from ACROS Organics (Fair Lawn, NJ, USA). Acetone used in CNIM fabrication was purchased from Sigma Aldrich (St. Louis, MO, USA). Multiwalled carbon nanotubes (MWCNTs) were sourced from Cheap Tubes Inc., Brattleboro, VT. The average diameters of the CNTs were ~30 nm and a length range of 15 μm. Flat composite PTFE membrane supported with polypropylene nonwoven fabric (Advantec MFS, Dublin, CA, USA; item number J020A330R, 129 µm thick, 0.2 µm pore size and 70% porosity) was used for CNIM fabrication and as control membrane. Simulated high fouling humic acid solution was prepared by dissolving humic acid salt in distilled water to stimulate fouling.

### 2.2. Membrane Preparation

Polytetrafluoroethylene membrane was used as the control membrane. The CNT immobilized membrane was prepared using a polytetrafluoroethylene (PTFE) laminate supported on the polypropylene composite membrane. The CNTs dispersion was carried out following previous papers published by this group [11,42]. Polyvinylidene difluoride (PVDF) was used as a binder during immobilization of the CNTs on PTFE membrane surface. The PVDF-nanotube dispersion was applied uniformly with a dropper over the membrane held on a flat surface to form the CNIM. The wet CNIM was kept under the hood for overnight drying.

### 2.3. Experimental Set-Up

**Figure 1:**
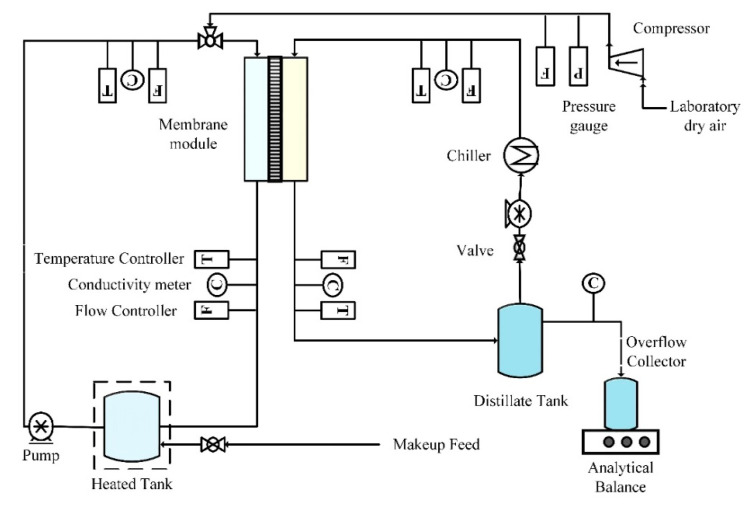
Experimental set up of the AS-MD for HA rejection.

In this study, direct contact membrane distillation (DCMD) configuration was used with modified air sparging technique. It was named as AS-MD (air sparged membrane distillation). AS-MD experiments were conducted using a closed-loop bench-scale membrane test unit (Figure 1). The disk-shaped module had an inner diameter of 4.2 cm and an active contact area of 13.85 cm^2^. Peristaltic pump (Cole Parmer, model 77200-52, Vernon Hills, IL, USA) was used to circulate feed water mixture through the membrane module and was recycled. The sparged air velocity was measured by a digital flow meter (Kelly Pneumatics, Inc., Newport Beach, CA, USA). The heating element and the temperature sensor (K-type, Cole Parmer) were connected to a temperature control unit that was used to regulate the temperature of the feed solution. The overflow of permeate water was collected as flux weighed on an analytical balance. Cold water was circulated in the condensing coil from a chiller to collect permeate vapor with recirculating permeate. Laboratory dry air supplied in the fume hood was sparged in the feed side. The electrical conductivity and turbidity of the distillate was continuously monitored using a portable pH/conductivity meter (Cole Parmer, Vernon Hills, IL, USA) and UV 2018.

### 2.4. Experimental Procedure

The performance of humic acid solution separation in air sparged MD were investigated in terms of temperature, concentration, and feed flow rate. The high fouling simulated humic acid solution was prepared by dissolving 100 mg HA in 1 L distilled water. The control PTFE and CNIM membranes were tested under various conditions, including volumetric concentration factor, temperatures (50, 60, and 70 °C), and feed flow speeds (45, 60, and 75 mL/min). For all the experiments, the distillate flow rate on the permeate side was kept steady at 1:1 ratio with feed. The water flux, HA rejection, and evaporation efficiency of the MD were evaluated in terms of the operational parameters.

The overall water flux can be calculated from Equation (1), where *W_water_* is the weight of water accumulated in the permeate side of the membrane at a given time *t* and *A* is the active membrane surface area.

(1) $$\mathrm{Water}\;\mathrm{Flux},\;J=\frac{W_{water}}{t*A}$$

Another important performance parameter of MD performance is the rejection %, expressed as Equation (2). It is denoted as the ratio between difference of feed and permeate concentration to the feed concentration.

(2) $$\mathrm{Rejection}\;(\%),\;R=\frac{C_{feed,\;in}-C_{permeate,\;t}}{C_{feed,\;in}}\times 100$$

Thermal efficiency of membrane distillation process is shown in Equation (3), where *Q_m_* is the heat transferred through the membrane by conduction and convection. *J_p_* is the flux in the permeate side, *A* is the active membrane surface area, and *H_v_* is the heat of vaporization for water.

(3) $$\mathrm{TE}\;(\%)=\frac{J_{p\;}A\Delta H_{v,w}}{Q_{m}}\times 100$$

Heat transferring through the membrane can be calculated by Equation (4), where *m_f_* is the feed flow rate and *C_p_* is the heat capacity of the water. To simplify the heat capacity, 100 mg/L HA is considered equal to pure water.

*Q_m_* = *m_f_*
*C_p_* (*T_f, in_* − *T_f, out_*) (4)

## 3. Result and Discussion

The following section explains the characterization of membranes and robustness of the air-sparged membrane distillation. The performance is evaluated in terms of flux, evaporation efficiency and rejection of HA molecules in the feed side. The mechanism of air sparged MD and fouling deposition reduction is duly explained.

### 3.1. Thermogravimetric Analysis

**Figure 2:**
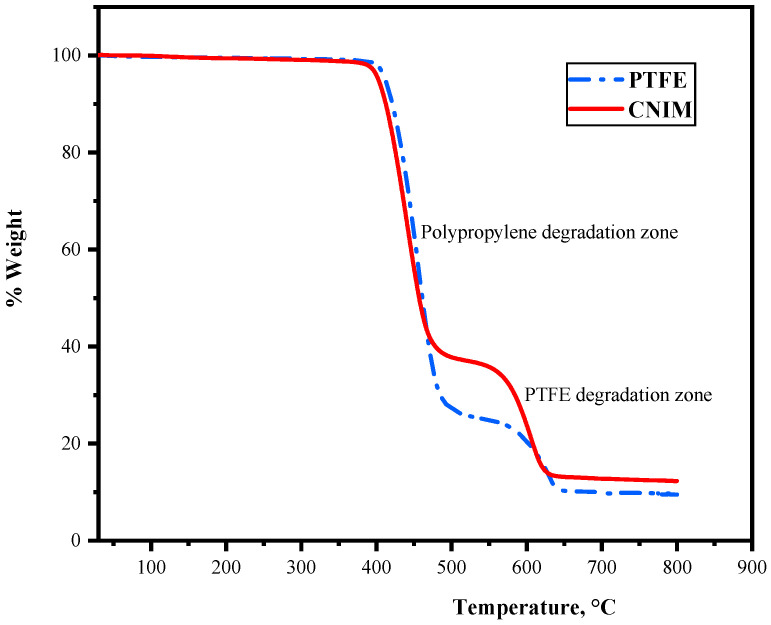
TGA curve for control PTFE and CNIM membrane.

Thermogravimetric analysis (TGA) was used to analyze the stability of the control PTFE membrane and CNIM at higher temperatures. The TGA curves are shown in Figure 2. It is observed that the initial thermal decomposition of the membrane began at 350 °C (degradation of the PP support layer), followed by the degradation of the PTFE active layer at 500 °C. The degradation characteristics of the membrane showed the applicability of the membranes at higher temperatures.

### 3.2. Porosity and Pore Size

Porosity of the hydrophobic membrane was an important property for flux in membrane distillation. The porosities of PTFE and CNIM were measured using the gravimetric method. The total molar gas permeation flux per unit transmembrane pressure difference across the porous membrane can be expressed as Equation (5).

(5) $$\frac{J_{i}}{\Delta P}=\frac{2}{3\;}\;(\frac{8RT}{\pi M}{)}^{0.5}\;\frac{1}{RT}\frac{r\in }{L_{p}}+\frac{P}{8\mu RT}\frac{\in r^{2}}{L_{p}}$$

where ε is surface porosity, *r* is mean pore radius (m) of the membrane, *μ* is the gas viscosity (Pa-s), *L_p_* is effective pore length, 𝑝 is the mean pressure, *M* is the molecular weight of gas, *R* is the gas constant (J K^−1^mol^−1^), and *T* is temperature (K). The gas permeation flux per unit driving force (*Ji/*Δ*P*) can be calculated as follows:

(6) $$\frac{J_{i}}{\Delta P}=\frac{N_{t,i}}{A_{t}}$$

where Δ*P* is the transmembrane pressure difference across the membrane area *A_t_*. *N_t,i_* is total molar gas permeation rate (mol s^−1^). The total gas permeation rate through the membrane was measured by a bubble flow meter. From Equation (5), the mean pore size (*r*) and the effective surface porosity over pore length, ε/*L_p_*, can be calculated from the slope (*S*_0_) and the intercept (*I*_0_) as follows:

(7) $$r=\frac{16}{3\;}\;(\frac{S_{0}}{I_{0}})(\frac{8RT}{\pi M}{)}^{0.5}$$

The modification of membrane surface by a small amount of CNTs did not alter the membrane morphology much. In a gas permeation test, the effective surface porosity over pore length ($\frac{\in }{L_{p}})$ of PTFE membrane and CNIM membrane were similar.

### 3.3. Contact Angle

**Figure 3:**
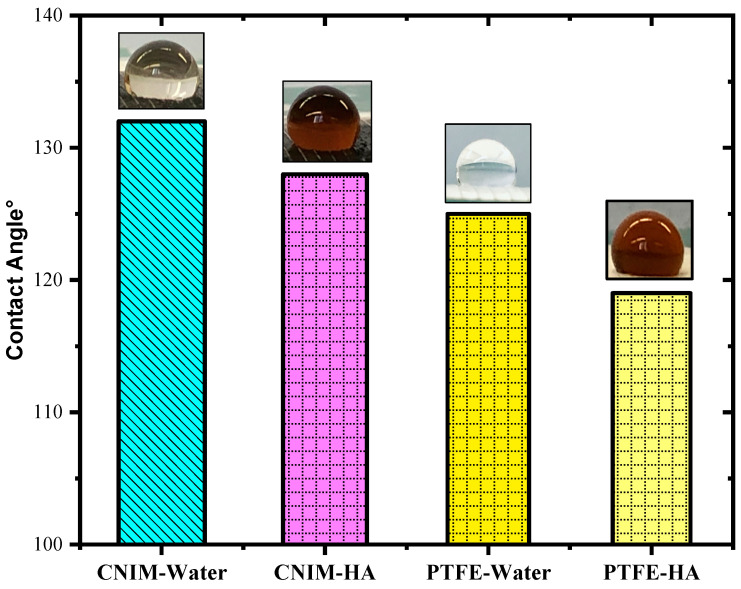
Contact angle of the membranes for water and humic acid solution.

The contact angles of DI water and 100 mg/L aqueous HA solution on the membrane surface, along with their images, are presented in Figure 3. The presence of CNTs resulted in the alteration of the contact angle of the drops on the PTFE membrane. In the case of deionized water, the contact angle for the plain PTFE membrane was 125° which decreased to 119° for the 100 mg/L HA solution. While for the CNIM membrane the DI water contact angle was 132° and 128°, for the HA solution it was at room temperature. The reduction in the contact angle for humic acid solution in CNIM attributed to the functionality of carbon nanotubes. The finding of the experiment was that the incorporation of CNTs increased the contact angle for water, rendering higher hydrophobicity, which indicated lesser wetting of the membrane surface.

### 3.4. Flux and Evaporation Efficiency in PTFE Membrane

**Figure 4:**
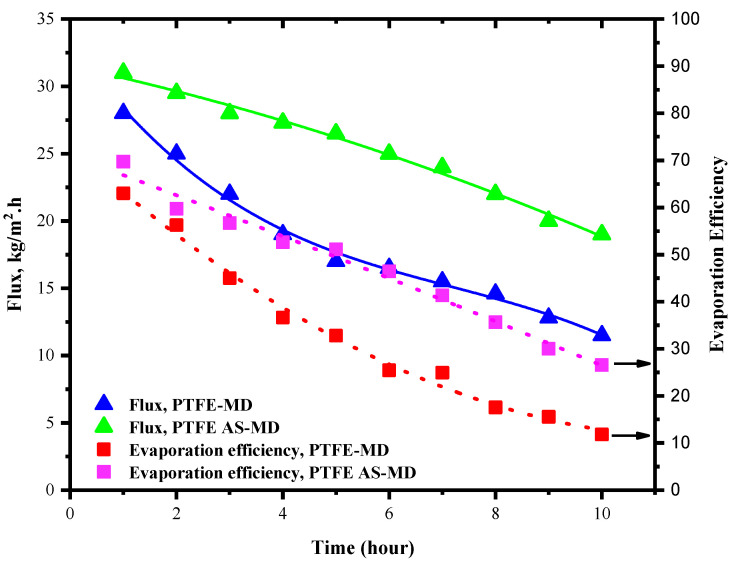
Flux and evaporation efficiency of PTFE control membrane. Feed HA concentration: 100 mg/L, temperature 60 °C, feed flow rate 75 mL/min.

The flux and evaporation efficiency for control PTFE membrane in regular and air sparged mode is shown in Figure 4. The concentration of the HA was kept at 100 mg/L and the experiment was run for 10 h. In case of regular membrane distillation shown in Figure 4, the flux of the PTFE membrane was found to be 28 kg/m^2^⋅h for the first hour; after that, the flux started declining with time. This is due to the deposition of HA layer over the membrane surface. The deposition amount and propensity increased with time. After running for a cycle of 10 h the flux declined by 60% of its initial value and finally reached a value of 12 kg/m^2^⋅h. In the meantime, the evaporation efficiency was also affected by the lesser vaporization and vapor molecules available for permeation. As a result, the evaporation efficiency declined from 72.3% to 10.12%. This steep decline in evaporation efficiency could be the impact of reduced vapor permeation through the membrane to the permeate side and possible heat loss because of fouling.

Meanwhile, the introduction of air sparging changed the behavior of the system and fouling deposition. Here, the idea was to utilize the effect of air sparging in reducing the fouling deposition. Throughout membrane distillation, supplementary HA solution was added to the system after each measurement of flux to maintain the HA concentration. In air sparged MD the water flux showed slight increment even though the flux declined after 10 h of operation as in regular MD but in different degrees. Initially in AS-MD the flux was 31 kg/m^2^⋅h which reduced by 39% to reach 21 kg/m^2^⋅h after 10 h. Overall, the air sparged MD showed 21.1% lesser drop in flux compared to regular MD.

### 3.5. Flux and Evaporation Efficiency for CNIM Membrane

**Figure 5:**
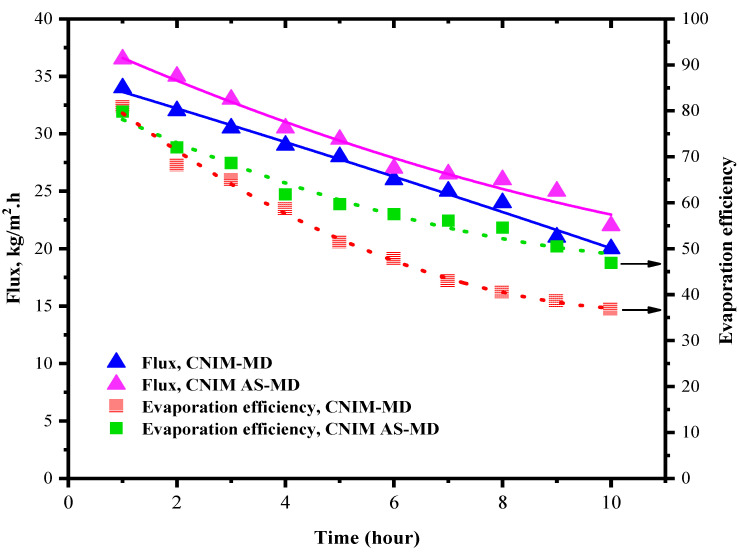
Flux and evaporation efficiency of 100 mg/L HA solution on CNIM membrane.

The generated water flux and evaporation efficiency for the CNIM membrane for both in regular MD and AS-MD are presented in this section in Figure 5. The experiments were run for 10 h like PTFE, and the fluxes were monitored. The introduction of CNTs on the membrane surface increased the water flux by as much as 13%. This is due the fact that the CNTs actively transport the water vapor molecules from feed side to the permeate side. The presence of CNTs on the membrane surface caused the deposition of the HA in membrane surface to reduce comparatively. As a result, the temperature polarization reduced ensuring higher heat transfer. In CNIM membrane, after 10 h the flux reduced by 41.1% which is 19% lesser than that of the control PTFE membrane. In the meantime, the evaporation efficiency was found to be 81% for CNIM membrane and reduced to 36% after 10 h due to fouling. Compared to the control PTFE membrane, the evaporation efficiency dropped down by 26%, showing lower deposition on the membrane surface.

In case of CNIM membrane for air sparging mode the relative reduction in flux for 10 h of operation was 33.3% and the evaporation efficiency dropped down by 42% from its initial value. In comparison to regular MD using control PTFE membrane, the end flux after 10 h AS-MD is 27% higher. This lesser flux reduction could be due to the breakdown of fouling layer and enhanced temperature distribution.

### 3.6. Effect of Temperature on Flux and Evaporation Efficiency

**Figure 6:**
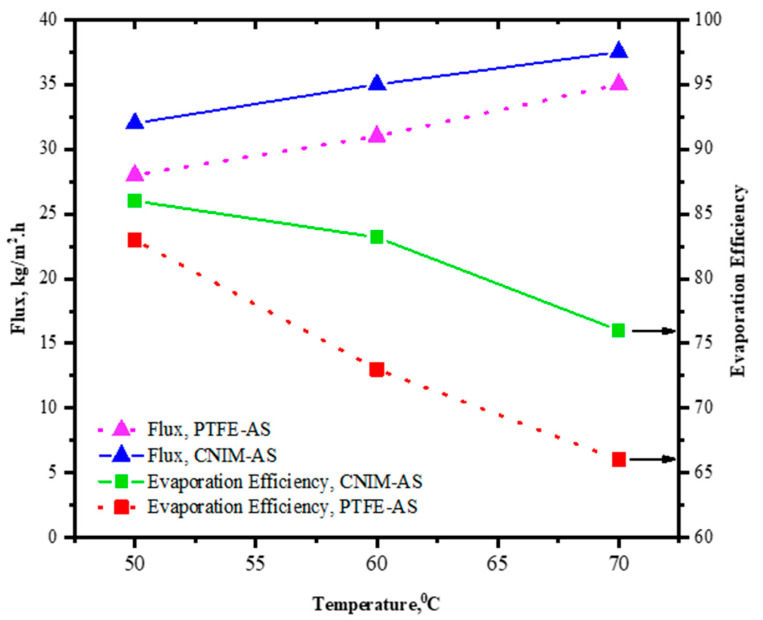
Effect of temperature on flux and evaporation efficiency for HA acid on control PTFE and CNIM.

The effect of temperature change on flux and evaporation efficiency (EE) is evaluated and shown in Figure 6. The experiments were performed at 50, 60, and 70 °C temperature for both the control PTFE and CNIM membrane with regular and air sparging modes of MD. Overall, the flux increased for increasing temperature for both the PTFE and CNIM membrane in either condition, whereas the EE showed opposite trend to flux. The evaporation efficiency declined as the temperature increased, which could be due to the loss of heat from the system at higher temperature. For a temperature change of 20 °C from 50 to 70 °C the flux of PTFE membrane with air sparging reached 35 kg/m^2^⋅h from 28 kg/m^2^⋅h, while for CNIM membrane the change was 32 to 37.5 kg/m^2^⋅h. However, the EE for CNIM membrane dropped by 11.6% for temperature change and for PTFE membrane it was 20.4%.

### 3.7. Flux and Evaporation Efficiency for Various Volumetric Concentrating Factor

**Figure 7:**
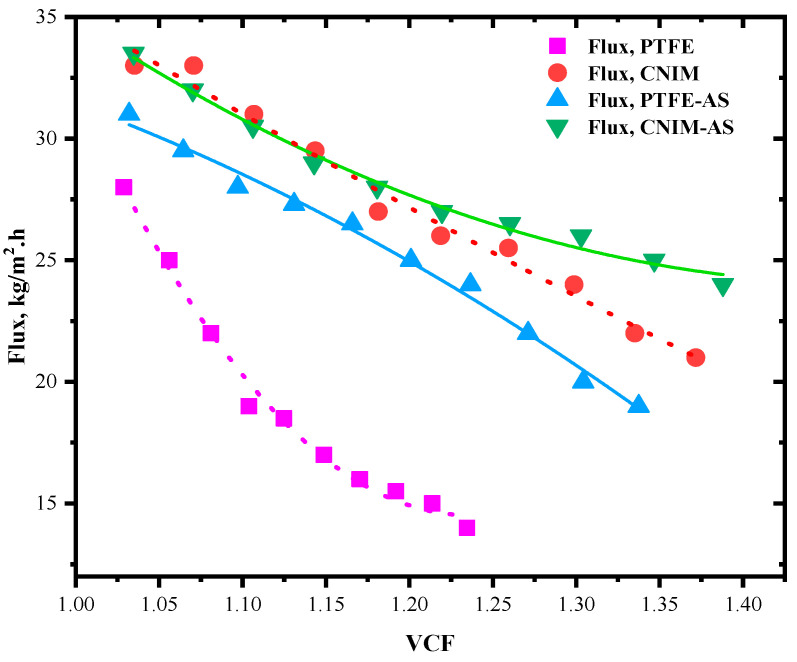
Variation of flux for various volumetric concentrating factor (VCF).

The trend of flux and evaporation efficiency depends on the concentration of the HA solution. If the concentration is increased, the subsequent effects are visible in their flux values. Here, the concentration effect is expressed as volumetric concentration factor. The VCF is defined as follows [43]:

(8) $$\mathrm{VCF}=\frac{V_{0}}{V_{0}-J_{0-t}}$$

where *V*_0_ is the initial feed volume (m^3^) and *J*_0*−t*_ is the total flux at a given time *t*. The effect of volumetric concentration factor on permeate flux is shown in Figure 7. This VCF is calculated for the 10 h of MD run after each flux calculation point. The feed was concentrating as long as the vapor permeated through the membrane, and no makeup water was added. Since the CNIM membrane actively transports water vapor molecules, the VCF was higher for CNIM based regular MD or AS-MD. Meanwhile, the flux reduction was lesser for CNIM membranes. The VCF was highest in case of AS-MD in CNIM membrane and it followed CNIM-AS > CNIM > PTFE-AS > PTFE trend. The flux reduction was minimum for AS-DCMD in CNIM membrane and maximum for PTFE membrane. In AS-MD at higher VCF the shear stress could eventually broke down the fouling layer and helped maintain a reasonable vapor pressure gradient to generate permeate flux. Figure 7 shows the individual flux trends for all the membrane and MD configuration modes. PTFE membrane showed heavy flux decline even at VCF of 1.24 while the CNIM based air spared membrane distillation maintained reasonable flux around VCF of 1.40. This shows the effectiveness of the air sparging in fouling removal during membrane distillation.

### 3.8. Mass Transfer Coefficient 

The overall mass transfer co-efficient is expressed by the following relationship. Here, *V* is the volume of the feed taken at time *t* = 0 and A is the effective membrane area. *C*_0_ is the initial concentration, *C* is the final concentration of HA, and *t* is time of MD run.

(9) $$k=\frac{V}{At\;}\;\left(1-\frac{C}{C_{0}}\right)$$

**Figure 8:**
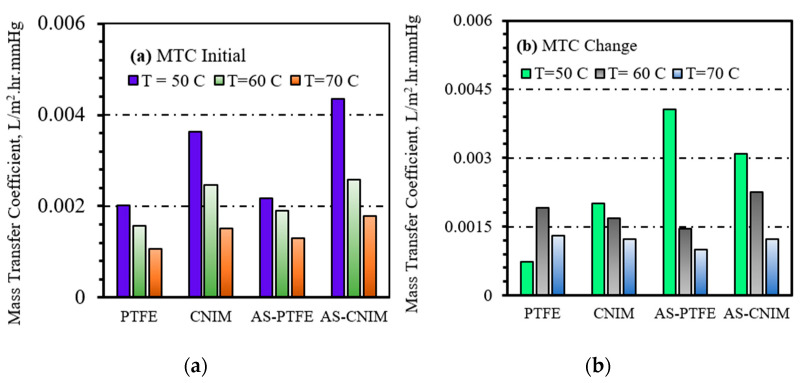
Mass transfer coefficient (MTC) for MD and AS-MD for control PTFE and CNIM membrane. (**a**) MTC initial. (**b**) Change in mass transfer coefficient in membrane -HA system.

Figure 8a represents the overall mass transfer co-efficient of the HA separation process. The mass transfer co-efficient of air sparged MD was 0.0044 L/m^2^⋅h⋅mmHg at the temperature of 50 °C for CNIM membrane. As the separation was carried out in higher temperature the mass transfer co-efficient decreased, even though the flux was increasing. The mass transfer co-efficient for control PTFE membrane in DCMD and AS-MD were similar due to lack of active transport sites in PTFE membrane. The inverse relationship of mass transfer coefficient with temperature was analogous for both PTFE and CNIM membrane.

On the other hand, the mass transfer co-efficient change after 10 h of operation was highest for PTFE membrane in air sparged membrane distillation. This is due to the fouling deposition on the membrane surface hindering the vapor permeation and inducing temperature polarization. In case of carbon nanotube immobilized membranes, the fouling deposition was lesser due to screening effect for both regular and air-sparged mode, that is why the change also smaller than the PTFE membrane. The change in MTC for different temperatures is shown in Figure 8b.

### 3.9. Reduction of HA Layer on the Membrane Surface

**Figure 9:**
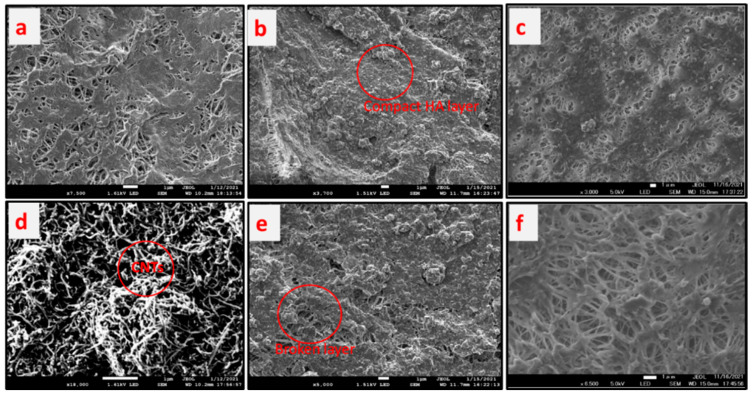
Scanning electron micrograph of membranes and fouling layer. (**a**) Pristine PTFE membrane. (**b**) Humic acid layer over PTFE. (**c**) Thin layer of humic acid in air sparging MD. (**d**) Carbon nanotube immobilized membrane (CNIM). (**e**) Broken humic acid layer over the CNIM membrane. (**f**) Cleaned CNIM surface with air sparging.

The fouling of membrane surfaces and its clean up using air sparging during membrane distillation is explained in this section. Figure 9 shows the scanning electron micrograph photos of the membrane surfaces at various conditions, while Table 1 summarizes fouling deposition per unit membrane area and flux reduction. Figure 9a,d show the pristine PTFE membrane used as control and CNIM membrane, respectively. The CNTs are dispersed over the membrane surface as shown in Figure 9d. Meanwhile, Figure 9b,c depict the fouled PTFE membrane by HA solution for regular and air sparged mode, respectively. The deposition on the membrane surface for regular DCMD 1.82 mg/cm^2^ and it reduced to 1.28 mg/cm^2^ in air sparged DCMD for PTFE membrane. On the other hand, for CNIM membrane the deposition reduced by 34.1% from 1.64 mg/cm^2^ to 1.08 mg/cm^2^. The relative reduction in fouling also seen from the flux value Table 1, where air sparging showed significant improvement.

### 3.10. Permeate Quality in the Presence of Humic Acid

**Figure 10:**
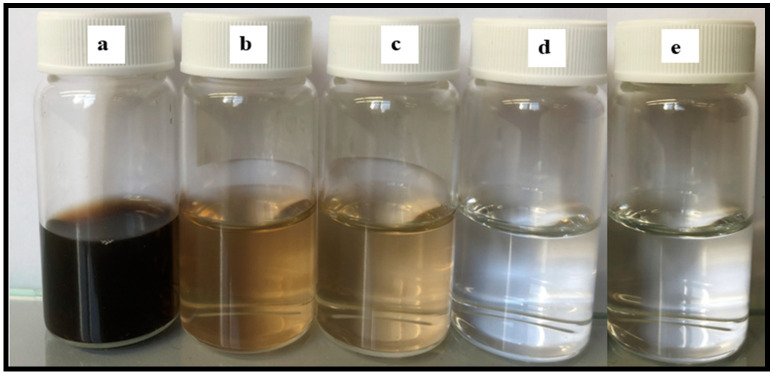
Rejection of HA solution and permeate turbidity: (**a**) feed humic acid solution of 100 mg/L. (**b**) Permeate of PTFE, MD. (**c**) Permeate of PTFE, AS-MD. (**d**) Permeate of CNIM, MD. (**e**) Permeate of CNIM, AS-MD.

**Figure 11:**
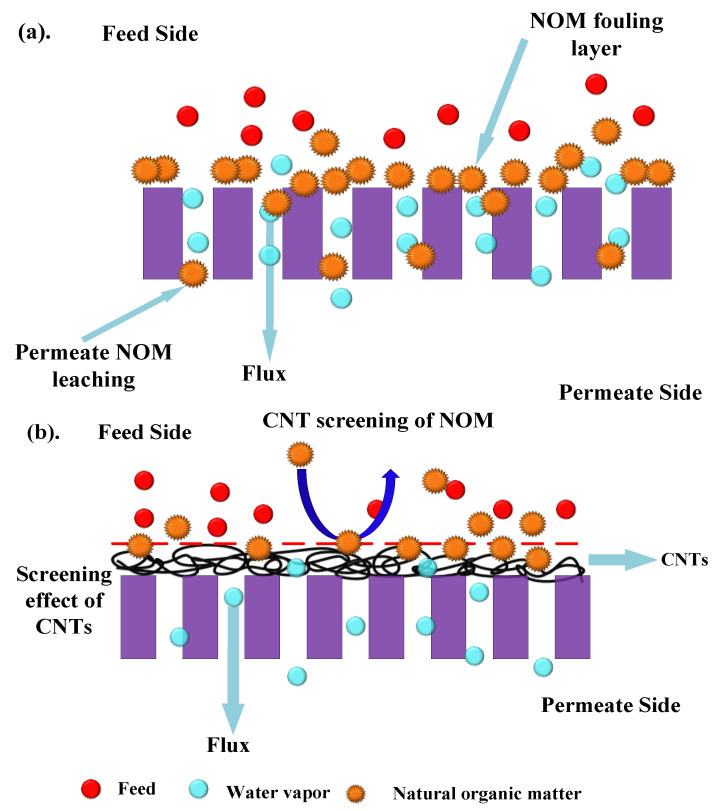
Mechanism: (**a**) Leaching of NOMs through the PTFE membrane pore. (**b**) Screening mechanism of CNTs to prevent NOMs leaching.

It was observed that the air sparging decreased the fouling deposition on the membrane surface. Figure 10 shows the permeate water after different modes of MD run. The permeate flux seemed to be largely affected by the volumetric concentration factor and this showed decreasing trends for all the membranes. When the concentration factor varied from 1.0 to 1.38, the ratio of permeate flux without and with air sparging enhanced from 1.20 for control PTFE to 1.71 for CNIM. With the concentration factor increasing, both the feed viscosity and the boundary layer thickness increased, which would aggravate concentration polarization on the membrane surface and cause the permeate flux to decline in some extent. The extensive fouling in the membrane surface and pores influenced leaching of foulants from feed side to the permeate side in PTFE membrane (Figure 11a). Therefore, the permeate water had some color after extended time of MD run.

Meanwhile, the addition carbon nanotubes changed the colloidal behavior of humic acid macromolecules by screening effect. The screening mechanism is shown Figure 11b. Here, the carbon nanotubes screen the humic acid molecules and do not let them deposit or clog the membrane surface. As a result, the leaching from the pores is prohibited.

The permeate conductivity was also checked from time to time for water quality. The conductivity was found in the range of 6–15 µS, which is a characteristic of pure water. Overall, the permeate quality improved by the dual action of CNIM and air sparging.

## 4. Conclusions

Commercial PTFE and CNIM membrane were characterized and applied in the treatment of high fouling HA solution in air sparged conditions. The porosity did not alter as much for CNIM membrane as was evident from the gas permeability test. The contact angle of the control membrane increased upon fabricating with carbon nanotubes rendering higher hydrophobicity. AS-MD was found to be more effective in the treatment HA solution for longer operating cycles. In air sparged conditions the deposition over the membrane surface was lesser compared to non-air sparging condition. The thermal efficiency was also higher in AS-MD mode. The higher flux in CNIM could be due to facilitated transport of water vapor molecules on the carbon nanotube surface. Meanwhile, lesser fouling is seen in AS-MD; this could be due to the exerted shear stress on the membrane surface by sparged air.

## Figures and Tables

**Table 1:** Fouling deposition and relative reduction in flux for PTFE and CNIM membrane.

System	Relative Flux Reduction %, DCMD	Relative Flux Reduction %, AS-DCMD	Deposition DCMD, (mg/cm^2^)	Deposition As-DCMD, (mg/cm^2^)	Decrease in Deposition, %
PTFE-HA	50	38.7	1.82	1.28	29.7
CNIM-HA	36.3	28.3	1.64	1.08	34.1

## Data Availability

Not applicable.
